# Direct Axillary Artery Cannulation as Standard Perfusion Strategy in Minimally Invasive Coronary Artery Bypass Grafting

**DOI:** 10.3390/jcdd12010031

**Published:** 2025-01-18

**Authors:** Christian Sellin, Ahmed Belmenai, Volodymyr Demianenko, Marius Grossmann, Hilmar Dörge

**Affiliations:** Department of Cardiothoracic Surgery, Heart-Thorax Center, Klinikum Fulda, University Medicine Marburg, Campus Fulda, 36043 Fulda, Germany; ahmed.belmenai@klinikum-fulda.de (A.B.); demcardio@gmail.com (V.D.); marius.grossmann@klinikum-fulda.de (M.G.); doerge@klinikum-fulda.de (H.D.)

**Keywords:** minimally invasive cardiac surgery, coronary artery bypass grafting, CABG, cardiopulmonary bypass, peripheral cannulation

## Abstract

Objective: Cardiopulmonary bypass (CPB) via the right axillary artery (RAA) has become an alternative perfusion strategy, especially in complex aortic procedures. This study delineates our technique and outcome with direct axillary cannulation utilizing the Seldinger technique, which we adopted as the standard perfusion strategy in the sternum-sparing minimally invasive total coronary revascularization via left anterior thoracotomy (TCRAT) using CPB. Methods: From November 2019 to December 2023, a total of 413 consecutive patients underwent nonemergent isolated coronary artery bypass grafting (CABG) via left anterior minithoracotomy on CPB with peripheral cannulation via the RAA and cardioplegic cardiac arrest, using this technique as a default strategy in the daily routine. All patients had multivessel coronary artery disease. The primary outcome was intraoperative cannulation-related complications (bleeding, revision, ischemia, wound healing complications). The secondary outcome was cannulation-related events during follow-up (blood pressure differences, incidence of brachial plexus injury, clinical signs of circulatory problems of arm and hand, re-interventions). Mean midterm follow-up was 18.7 ± 12.3 [1.1–51.2] months. During follow-up, 16 patients died. Overall, a total of 397 patients (344 male; 67.6 ± 9.7 [32–88]) were included for follow-up (100%). Results: The RAA was successfully cannulated in 100% of patients. A cannula size of 16 Fr was used in 34.6%, 18 Fr in 63.9% and 20 Fr in 1.5% of all patients. There was no intraoperative bleeding complication. In two patients, intraoperative revision of the RAA was required, necessitating a venous patch repair. At follow-up, there were no differences between the systolic and diastolic blood pressure or the pressure gradients between the right and left arm. Transient numbness of the right hand was observed in two patients. Permanent numbness was not observed. No patient needed further intervention or surgical revision of the RAA. Conclusions: The right axillary cannulation is feasible and safe in terms of vascular injury and brachial plexus injury with excellent in-hospital and follow-up outcome.

## 1. Introduction

The ascending aorta is the most common site of arterial cannulation for cardiopulmonary bypass (CPB) in cardiac surgery [1,2]. In the presence of ascending aortic aneurysms, aortic dissections or severe atherosclerosis, an ascending aortic cannulation can be dangerous and risky [1,2,3]. Furthermore, the growing prevalence of minimally invasive techniques in cardiac surgery using small sternum-sparing incisions has led to an increased utilization of peripheral cannulation for CPB. In this context, wire skills are becoming increasingly important to ensure a safe procedure. In such cases, the femoral artery is the most frequently chosen alternative site for arterial cannulation. However, this cannulation site also has disadvantages and risks in case of concomitant vascular diseases as often prevalent in coronary heart disease, the risk of retrograde embolization or in dissections extending into the inguinal vessels [1].

CPB via the right axillary artery (RAA), first reported by Villard et al. [4] in 1976, has become an alternative perfusion strategy, especially in acute aortic dissections, redo aortic surgery or in patients with severe aortic atherosclerosis [5]. The main advantages of this perfusion strategy compared to other cannulation strategies are the preservation of antegrade flow through the aortic arch, antegrade cerebral perfusion, and minimization of aortic manipulation [6,7,8,9,10].

Different axillary cannulation techniques have been described with corresponding advantages and disadvantages. Nonetheless, despite the potential advantages of direct axillary cannulation, the literature regarding its safety is limited [6,7,8,9,10,11]. A major risk for direct axillary cannulation is the potential for vascular injury and iatrogenic dissections. Overall, the complication rate during direct axillary cannulation is reported to be 1 to 10% [5]. Various studies [6,7] analyzed the side-graft technique for axillary cannulation and found a lower complication rate compared to the direct cannulation technique. Pitfalls of the side-graft technique are anastomotic bleeding [7,11], longer procedure times [7], possible hyperperfusion of the ipsilateral arm [12] or permanent retention of foreign material at the end of the operation [6].

Excellent outcomes and low complication rates using the Seldinger technique [13] were reported for direct axillary cannulation in acute type A dissection procedures [11].

Minimally invasive coronary bypass grafting, especially in patients with generalized atherosclerosis, calcifications in the descending aorta, and peripheral vascular disease, presents unique challenges. The selection of the cannulation site should be guided by preoperative CT scans to minimize complications [14,15]. Nonetheless, this selection process is inherently susceptible to potential mistakes.

This study presents our surgical technique and focuses on the outcome of direct axillary cannulation using the Seldinger technique as a standard perfusion strategy for CPB in minimally invasive coronary on-pump surgery.

## 2. Methods

### 2.1. Patient Selection and Data Collection

From November 2019 to December 2023, a total of 413 consecutive patients underwent nonemergent isolated CABG via left anterior minithoracotomy on CPB with peripheral cannulation via the RAA and cardioplegic cardiac arrest (transthoracic aortic cross-clamping) in our institution. This technique was employed as a default strategy in our daily routine practice for sternum-sparing minimally invasive coronary revascularization.

All patients were scheduled after discussion with the heart team [16] including a recommendation, according to guideline indications [17], on which coronary arteries should be grafted.

Emergency patients (meaning catheterization and operation on the same day), patients with significant atheromatous disease of the ascending aorta, patients with moderate or severe aortic regurgitation, and patients undergoing reoperation were excluded.

During follow-up period, 16 patients died. Overall, a total of 397 patients were included for follow-up (100%) to investigate the outcome of direct cannulation of the RAA using the Seldinger technique for minimally invasive on-pump CABG.

Data are part of our internal quality assurance documentation and were retrospectively extracted from patient records and are presented as mean (±standard deviation) or number (percentage). The follow-up data were collected prospectively through telephone interviews, using a structured questionnaire by a study nurse, acquisition and evaluation of medical findings and physical examinations. Statistics were calculated with SPSS (Version 29.0.0.0, IBM SPSS Statistics, Armonk, NY, USA).

### 2.2. Preoperative Evaluation

All patients underwent a CT angiography in addition to the standard institutional preoperative examinations to screen the ascending aorta, the aortic arch and major arterial branches for atherosclerotic disease and anatomical abnormalities.

### 2.3. Anesthesia

Standard cardiac anesthesia techniques were used for the induction and maintenance of anesthesia. All patients were intubated with a single-lumen endotracheal tube (Rüschelit Super Safety Clear, Teleflex Medical, Westmeath, Ireland). Invasive monitoring was performed using venous lines and standard arterial lines on the left and the right arm to measure the blood pressure and to analyze the blood pressure curve. In all patients, transoesophageal echocardiography (TOE) was performed to assist positioning the guidewires for femoral venous cannulation.

### 2.4. Surgical Technique

The surgical technique of total coronary revascularization via left anterior minithoracotomy has been described in detail in recent studies [15,18]. The present publication focuses on the precise description of our routine strategy for CPB using peripheral right axillary artery cannulation.

The operations were performed in supine position.

**Figure 1:**
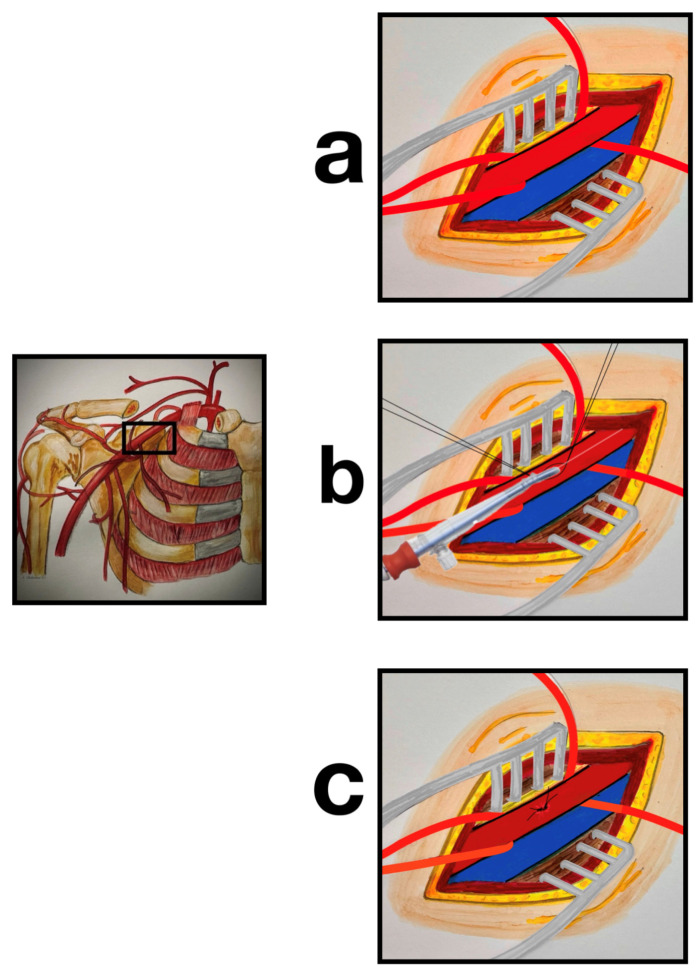
Schematic representation of the axillary cannulation technique: (**a**) preparation of the right axillary artery and vein, sling of the right axillary artery; (**b**) two purse-string sutures with 5-0 Prolene were placed in an oval shape on the top of the artery for direct cannulation of the right axillary artery using the Seldinger technique; (**c**) closure of the cannulation site using the purse-string sutures provided.

Peripheral arterial cannulation for CPB was performed via the RAA. A small skin incision about 2–3 cm was made approximately 1 cm below the middle-to-lateral part of the clavicle. The pectoralis major muscle was separated along the direction of its fibers. The underlying pectoralis minor muscle was retracted laterally and inferiorly. The RAA was exposed over a distance of about 2 cm and pulled up by a looped silicone tape (Figure 1a). The desired cannulation site was as proximal as possible in order to be able to use the largest possible cannula. Two purse-string sutures with 5-0 Prolene were placed on the anterior surface of the RAA in an oval shape in the longitudinal direction to prevent a vessel stenosis following knotting (Figure 1b).

After administration of 400 U/kg of heparin intravenously to achieve an activated clotting time (ACT) of more than 400 s, arterial cannulation was performed by puncturing the RAA within the two purse-sting sutures and inserting a Seldinger wire. After dilatation and short longitudinal incision of the vessel, the arterial cannula was inserted 3 cm up to a maximum of 4 cm (Figure 1b, Video). Three cannula sizes (16/18/20 Fr OptiSite Arterial Perfusion Cannula; Edwards Lifesciences, Irvine, CA, USA) were used, depending on the vessel size and the patient’s required cardiac output. Using the purse-string sutures provided, the cannula was fixed and additionally sutured at the skin level. Simultaneous, percutaneous venous cannulation was achieved through the common femoral vein. A venous cannula (23 Fr Bio-Medicus, Medtronic, Minneapolis, MN, USA) was placed in the right atrium guided by TOE.

At the end of CPB, the arterial cannula was removed from the RAA and the vessel was closed blood-tight using the purse-string sutures (Figure 1c). Following this, the analysis of the blood pressure curve on the right brachial or radial site, the blood flow in the right arm, and any existing pressure gradients compared to the left side were checked and analyzed.

### 2.5. Endpoints and Definitions

The primary outcome was intraoperative cannulation-related complications, including intraoperative bleeding, intraoperative revision of the RAA in case of vascular injury or iatrogenic dissection of the RAA and ischemia distal to the axillary cannulation site.

An axillary artery cannulation-related wound healing complication was defined as requirement for surgical exploration because of infection or seroma.

The secondary outcome was cannulation-related events during the follow-up period including difference between right/left-sided systolic and diastolic blood pressure and analysis of blood pressure gradients. Moreover, we investigated incidence of brachial plexus injury, clinical signs of circulatory problems of the right hand and arm, and the need for re-intervention or surgical revision of the RAA. Brachial plexus injury was defined as transient or permanent numbness or weakness of the right hand or arm based on neurological and neurophysiological (nerve conduction study) examinations which were carried out in the event of complaints.

### 2.6. Ethical Standards and Consent Statement

The Institutional Review Board (IRB) or equivalent ethics committee of the Philipps University of Marburg approved the study protocol and publication of data (23-173 RS).

The patients provided informed written consent for the publication of the study data.

## 3. Results

The study group consisted of 413 consecutive, nonemergent patients (360 male; 67.6 ± 9.9 [32–88] years). Mean EuroSCORE II was 3.0 ± 2.8. All patients had multivessel coronary disease, of which 32.2% had a left main stem stenosis. Baseline and clinical parameters are given in Table 1.

For CPB, the RAA was successfully cannulated in 100% of patients, and three different cannula sizes were used (OptiSite Arterial Perfusion Cannula; Edwards Lifesciences, Irvine, CA, USA). A cannula size of 16 Fr was used in 34.6%, 18 Fr in 63.9% and 20 Fr in 1.5% of all patients. Operative data are given in Table 2 including duration of operation, CPB and aortic cross-clamping.

**Figure 2:**
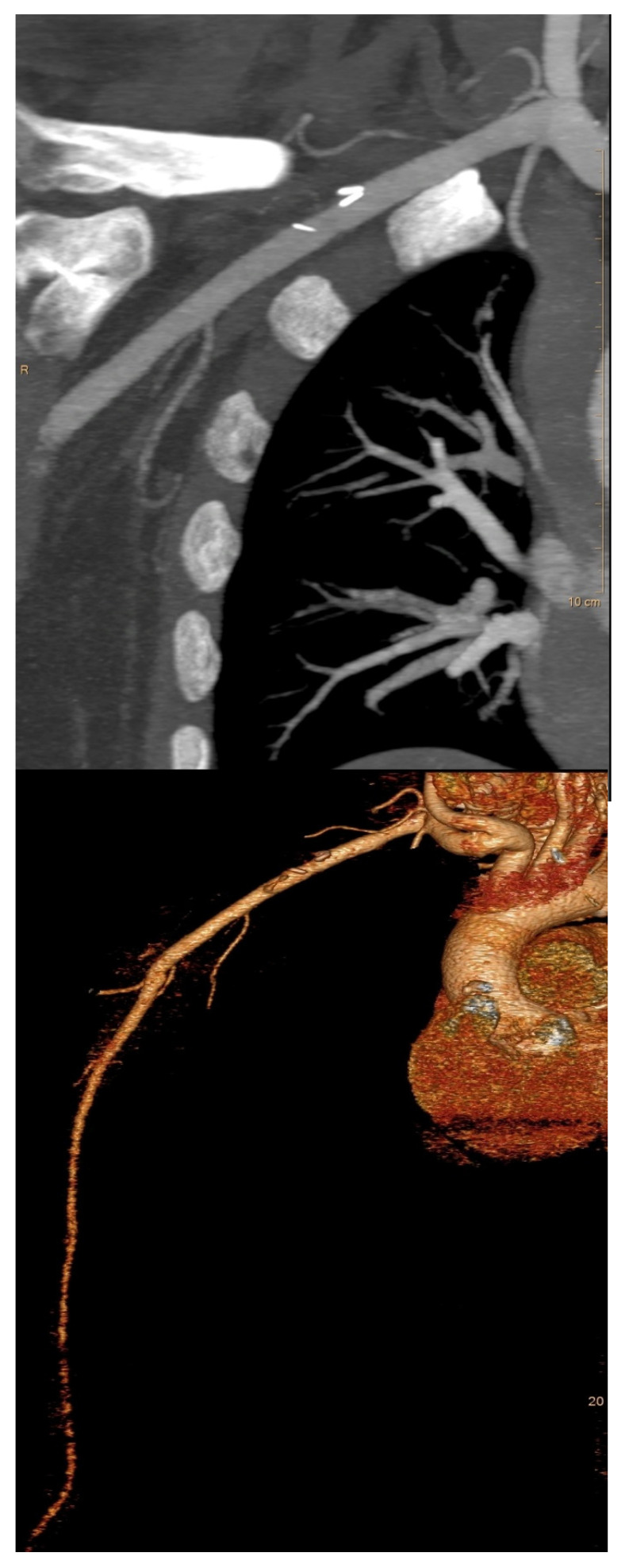
Postoperative CT scan after axillary cannulation and venous patch repair shows no stenosis of the vessel lumen after axillary cannulation.

There was no intraoperative bleeding complication related to the cannulation site. In two patients, an intraoperative revision of the RAA after completing of CPB using a venous patch repair was necessary to prevent a stenosis. One out of these two patients received a postoperative CT angiography to monitor the surgical result and blood flow to the arm (Figure 2).

One superficial wound healing complication was observed, specifically a seroma, necessitating surgical wound revision and secondary suturing. In-hospital complications associated with axillary cannulation are given in Table 3.

Mean follow-up was 18.7 ± 12.3 [1.1–51.2] months and was completed to 100%. Median of our follow-up was 15.8 months, and the interquartile range (IQR) was 17.7 months. The systolic and diastolic blood pressure values comparing the right and the left upper extremities showed no difference, as did the pressure gradients. The maximum measured pressure gradient was 24 mmHg.

Transient numbness of the right hand was observed in two patients; permanent numbness of the right hand was not observed during follow-up. No patient needed a further intervention or surgical revision of the RAA. Follow-up data are provided in Table 4.

## 4. Discussion

This study presents our technique and outcome of direct axillary cannulation using the Seldinger technique as standard perfusion strategy for CPB in sternum-sparing minimally invasive CABG. Our data show, based on a large patient cohort, that right axillary cannulation is feasible and safe in terms of vascular injury and brachial plexus injury with excellent in-hospital and follow-up outcome associated with the cannulation site.

Cannulation of the RAA for CPB is now commonly used as part of planned or emergency operations on the aorta, often involving the aortic arch, where an ascending aortic cannulation can be dangerous [1,3]. For an increasing number of minimally invasive cardiac surgical procedures, the femoral artery is often used for CPB, with limitations of stenosis or atheromatosis of the inguinal vessels or the disadvantage of retrograde body perfusion. To eliminate the disadvantages of femoral cannulation and to ensure optimal, antegrade perfusion of the body and especially the aortic arch in sternum-sparing, minimally invasive CABG via left anterior minithoracotomy, the right axillary artery appears to be the appropriate cannulation site.

Cannulation of the RAA has several advantages compared to aortic and femoral cannulation [1,19,20,21]. Cannulation and de-cannulation of the ascending aorta, and thus, the “sandblast” effect [22,23], a turbulent flow from the tip of the aortic cannula, can be largely avoided. Furthermore, in patients with coronary artery disease requiring surgery, there is an increased rate of patients with significant peripheral arterial disease, which can make femoral cannulation disadvantageous and difficult. In our cohort, more than 35% of patients had peripheral arterial disease, highlighting the importance of this point. In contrast, the RAA is invariably free of atheroma or severe calcification, making it an ideal site for arterial cannulation [23,24]. In addition, the possibility of antegrade perfusion of the aortic arch and the vessels supplying the brain and the associated avoidance of embolism through atherosclerotic debris is important with regard to the neurological outcome of the patient [19,24,25,26].

Different axillary cannulation techniques have been described and compared in various studies [1,2,3,19,25,27]. The direct cannulation technique of the axillary artery has been reported to be prone to complications. The incidence of vascular injuries as a consequence of direct axillary cannulation is reported to be between 2.8% and 9.0% [3,5,10]. In comparison with the side-graft technique, this is between 0.5% and 1.5% [6,7]. Similar data are also described for the rate of iatrogenic aortic dissections. Here, the incidence with direct cannulation is 1.4% to 4.1% compared to 0% with the side-graft technique [6,10]. In contrast, a relative hyperperfusion of the right arm using the side-graft technique can be a relevant problem, especially with a small diameter of the RAA. A possible consequence can be a less effective perfusion of the body [25]. Several studies [11,27] achieved very good results with direct axillary cannulation using the Seldinger technique in acute type A dissection operations and elective aortic surgery procedures. In the present study, intraoperative revision of the axillary cannulation site was necessary in two cases to avoid stenosis, i.e., 0.5%. Moreover, we observed no iatrogenic dissection of the RAA or the aorta and no hyper- or hypoperfusion of the right arm. Careful handling and wire skills are advisable to ensure a safe procedure.

Compared to arterial cannulation in the aortic position, there is an increased mean arterial line pressure with axillary cannulation. It depends on the BSA and the required cardiac output of the patient, the size of the RAA and the size and flow characteristics of the cannula used. In our study, we observed a mean arterial line pressure on average of 263 mmHg at 100% of the calculated cardiac output. So far, an adequately comparable data basis for this is rare in the literature. Hillebrand et al. [25] reported an average of the mean arterial line pressure during axillary cannulation of 324 mmHg. Various studies have discussed the insertion depth of the axillary cannula as a factor in this regard. Furthermore, this appears to have an important influence on the patient’s neurological outcome. Various authors recommend an insertion depth of 3–7 cm in order to avoid obstruction of the origin of the vertebral artery [5,25]. Taking these considerations into account, we inserted the axillary cannula 3 to a maximum of 4 cm in the RAA and chose the most anatomically proximal cannulation site to the RAA.

During the follow-up period, two patients (0.5%) with a transient numbness of the right hand were observed. With a very low rate of postoperative neurological dysfunctions, the present study is in line with the results of other studies [3,27,28].

Direct axillary arterial cannulation using the Seldinger technique is safe and associated with favorable outcomes in patients undergoing minimally invasive on-pump CABG.

## Figures and Tables

**Table 1:** Baseline and clinical parameters.

Variables	N = 413 N (%)
Age (years)	67.6 ± 9.9 (32–88)
≥80 years	54 (13.1%)
Male	360 (87.2%)
BMI (kg/m^2^)	28.4 ± 4.5 (18.0–42.6)
BMI ≥ 35	35 (8.5%)
BSA (m^2^)	2.02 ± 0.2 (1.5–2.7)
Calculated cardiac output (L/min)	4.8 ± 0.5 (3.5–6.5)
Diabetes mellitus	145 (35.1%)
Chronic lung disease	71 (17.2%)
Peripheral arterial disease	147 (35.6%)
EuroSCORE II (%)	3.0 ± 2.8 (0.4–29.6)
EuroSCORE II ≥ 4	101 (24.5%)
LVEF (%)	48.9 ± 10.0 (10–65)
LVEF ≤ 30	33 (8.0%)
2-vessel disease	96 (23.2%)
3-vessel disease	317 (76.8%)
Left main stenosis > 50%	133 (32.2%)
Recent NSTEMI	170 (41.2%)
Prior PCI	103 (24.9%)

Values are expressed as mean ± SD. Minimum–Maximum values are in parenthesis. BMI: body mass index, LVEF: left ventricular ejection fraction, BSA: body surface area, NSTEMI: non-ST-elevation myocardial infarction.

**Table 2:** Operative data.

Operative Data	N = 413 N (%)
Number of distal coronary anastomoses	3.1 ± 0.8 (2–5)
Duration of (minutes)	
• CPB	159 ± 41 (52–313)
• Aortic cross-clamping	99 ± 32 (22–255)
• Operation	330 ± 73 (145–705)
Cannula size	
❖ 16 Fr	143 (34.6%)
➢ BSA	2.0 ± 0.2 (1.5–2.4)
➢ Calculated cardiac output (L/min)	4.4 ± 0.3 (3.5–5.2)
➢ Mean arterial line pressure at 100% cardiac output	263 ± 52 (123–433)
❖ 18 Fr	264 (63.9%)
➢ BSA	2.0 ± 0.2 (1.5–2.7)
➢ Calculated cardiac output (l/min)	5.0 ± 0.4 (3.7–6.0)
➢ Mean arterial line pressure at 100% cardiac output	265 ± 55 (146–429)
❖ 20 Fr	6 (1.5%)
➢ BSA	2.1 ± 0.1 (1.9–2.2)
➢ Calculated cardiac output (l/min)	6.2 ± 0.2 (6.0–6.5)
➢ Mean arterial line pressure at 100% cardiac output	233 ± 34 (196–288)
Mean arterial line pressure at 100% cardiac output (mmHg)	263 ± 53 (123–433)

Values are expressed as mean ± SD. Minimum–Maximum values are in parentheses. CPB: Cardiopulmonary bypass.

**Table 3:** In-hospital complications associated with axillary cannulation.

Variables	N = 413 N (%)
Intraoperative cannulation-related events	
• Bleeding at cannulation site	0 (0.0%)
• Intraoperative revision of the RAA (venous patch repair)	2 (0.5%)
• Intraoperative aortic dissection	0 (0.0%)
• Intraoperative dissection of the RAA	0 (0.0%)
Perioperative CT angiography	1 (0.2%)
Wound healing complications at cannulation site	1 (0.2%)
• Superficial	1 (0.2%)
• Deep	0 (0.0%)

RAA: right axillary artery.

**Table 4:** Follow-up results.

Variables	N = 397	
Blood pressure		
Right hand		
• Systolic blood pressure (mmHg)	128 ± 15 (90–179)	
• Diastolic blood pressure (mmHg)	75 ± 10 (44–112)	
Left hand		
• Systolic blood pressure (mmHg)	129 ± 15 (92–176)	
• Diastolic blood pressure (mmHg)	75 ± 11 (44–115)	
Systolic pressure gradient (mmHg)	Right > Left	Left > Right
• ∆ gradient < 10	171 (43.1%)	155 (39.0%)
• ∆ gradient 11–20	28 (7.0%)	30 (7.6%)
• ∆ gradient 21–25	6 (1.5%)	7 (1.8%)
Feeling cold in the right hand	0 (0.0%)	
Brachial plexus injury		
• Transient numbness of the right hand/arm	2 (0.5%)	
• Permanent numbness of the right hand/arm	0 (0.0%)	
Re-intervention or surgical revision of the RAA	0 (0.0%)	

Values are expressed as mean ± SD. Minimum–Maximum values are in parenthesis. RAA: right axillary artery.

## Data Availability

The data presented in this study are available on request from the corresponding author.

## Abbreviations

BMI	body mass index
BSA	body surface area
CABG	coronary artery bypass grafting
CPB	cardiopulmonary bypass
IQR	interquartile range
LVEF	left ventricular ejection fraction
NSTEMI	non-ST-elevation myocardial infarction
PCI	percutaneous coronary intervention
RAA	right axillary artery
TCRAT	total coronary revascularization via anterior thoracotomy
TOE	transoesophageal echocardiography
